# The burden of firearm injuries on the hospital system, 2000–2020

**DOI:** 10.1186/s40621-023-00420-1

**Published:** 2023-03-01

**Authors:** Anis Davoudi, Lindsey Woodworth

**Affiliations:** 1 Department of Epidemiology, Johns Hopkins University, 615 N. Wolfe Street, Baltimore, MD 21205 USA; 2 Department of Economics, University of South Carolina, 1014 Greene Street, Columbia, SC 29208 USA

**Keywords:** Firearms, Hospitals, USA, Diagnosis codes

## Abstract

**Background:**

Firearm injuries are a long-running yet preventable public health concern in the USA. We analyzed national inpatient data to determine the burden of firearm injuries on the USA hospital system. For each year from 2000–2014 and 2016–2020, we calculated the annual frequency of firearm hospitalization in the USA overall and by the intent of the shooter. We also calculated the rate of firearm hospitalizations per 100,000 inpatient encounters. For each outcome, we used regression analysis to estimate the average year-over-year change. Finally, we explored the types of firearms responsible for firearm hospitalizations.

**Findings:**

Each year during 2000–2020 (excluding 2015), there were an average of 30,428 firearm hospitalizations in the USA. On average, firearm hospitalizations represented 84 out of every 100,000 inpatient encounters each year. There was not a statistically significant year-over-year increase in firearm hospitalizations for either the periods 2000–2014 or 2016–2020. However, firearm hospitalizations were noticeably higher in 2020 than in other years. Until 2019, the most frequent intent among firearm hospitalizations was assault. Beginning in 2019, assaults were outnumbered by unintentional firearm hospitalizations. According to diagnosis codes, handguns were used more often than rifles/shotguns/larger firearms in firearm injuries that resulted in hospitalization for the intents assault (27.93% handguns; 5.87% rifles/shotguns/larger firearms), unintentional (23.94% handguns; 10.48% rifles/shotguns/larger firearms), self-harm (46.63% handguns; 14.35% rifles/shotguns/larger firearms) and undetermined (17.82% handguns; 6.21% rifles/shotguns/larger firearms). Frequently, the type of firearm responsible for the hospitalization was not recorded in the patient’s diagnosis code.

**Conclusion:**

Firearm injuries inflict a significant burden on the hospital system in the USA. While firearm hospitalizations were unusually high in 2020, there is not strong evidence that the burden of firearm injuries on the hospital system is changing over time. The frequent non-identification of the type of firearm responsible for the injury in hospital patients’ diagnosis code complicates injury surveillance efforts.

## Introduction

In 2020, over 45,000 people died in the USA from a firearm injury (CDC 2021). Annual firearm fatalities in 2020 were 58% higher than in the year 2000 (CDC 2021). The deadly nature of firearm injuries suggests a burden on the healthcare system. Hospitals, specifically, may bear the brunt of the burden of caring for individuals injured by firearms.

There are many reasons why firearm injuries occur, and previous research has shown that the majority of firearm hospitalizations are due to assault. Assaults represented 80% of gunshot wounds in Pennsylvania trauma centers from 2003 to 2015 (Gross et al. 2017), 74% of firearm hospitalizations in California in 1991 (Vassar & Kizer 1996), and 61.7% of firearm hospitalizations among young people nationally in 2009 (Leventhal et al. 2014). Understanding whether there are trends in shooters’ intents over time is important for policymaking.

Also important for policymaking is understanding the types of firearms responsible for injuries. In recent years, there have been numerous proposals to restrict access to certain types of guns. For instance, in 2022, the US House of Representatives passed legislation to reinstate a ban on semi-automatic guns (i.e., the “Assault Weapons Ban of 2022”). Others have documented that when the intent of the shooter is suicide, handguns are the most commonly used firearm (e.g., Hanlon et al. 2019; Wintemute et al. 1988). Yet, whether handguns are also chiefly used for assault is relevant for efforts to minimize violent crime.

This study’s aim was to estimate the annual burden of firearm injuries on the USA hospital system and to analyze the intents responsible for firearm hospitalizations (e.g., assault and self-harm). The study also aimed to explore the types of firearms responsible for the injuries.

## Methods

We used publicly available data from the National (Nationwide) Inpatient Sample (NIS) of the Healthcare Cost and Utilization Project (HCUP) on aggregate counts of each diagnosis code, each year, among inpatients. The NIS is constructed from HCUP’s State Inpatient Databases which contain all inpatient data provided to HCUP by the states, covering over 97% of the USA population.

Diagnosis codes may be used to reveal both shooters’ intents and firearm types for firearm injuries. We used the weighted, unduplicated counts of each diagnosis code which were published online by HCUP in spreadsheets (HCUP NIS, 2021). These counts were constructed by HCUP by applying a discharge weight to the NIS to reflect national estimates across all USA community hospitals, excluding rehabilitation and long-term acute care hospitals. HCUP’s deduplication corrected for instances in which the same diagnosis code was included twice within a single discharge record. HCUP masked diagnosis code counts when the count within a year-code was less than or equal to 50. When this was the case, the small unseen count was presumed to be zero in our analysis. We considered the years 2000–2014 and 2016–2020. Data were not reported in the online spreadsheets for 2015 because of the switch from International Classification of Diseases, Ninth Revision, Clinical Modification (ICD-9-CM) to International Classification of Diseases, Tenth Revision, Clinical Modification (ICD-10-CM).

We focused on diagnosis codes that indicated an injury due to a firearm. We identified the appropriate diagnosis codes using the CDC’s External Cause-of-Injury matrices. The portions of the ICD-9-CM and ICD-10-CM matrices that are relevant to our study (i.e., the parts that pertain to *firearm* injuries) are presented in Table 1. These matrices stratify diagnosis codes by the intent of the shooter. The five intents they delineate are (i) unintentional, (ii) self-harm, (iii) assault, (iv) undetermined, and (v) legal intervention or war. For ICD-10-CM codes, we only considered *initial* encounter diagnosis codes in our analysis.

**Table 1:** Firearm diagnosis codes from CDC’s external cause-of-injury matrices

Intent	ICD-9-CM (2000–2014)	ICD-10-CM (2016–2020)
Unintentional	E9220, E9221, E9222, E9223, E9228, E9229	W320XX, W321XX, W3300X, W3301X, W3302X, W3303X, W3309X, W3310X, W3311X, W3312X, W3313X, W3319X, W3400X, W3409X, W3410X, W3419X
Self-Harm	E9550, E9551, E9552, E9553, E9554	X72XXX, X730XX, X731XX, X732XX, X738XX, X739XX, X748XX, X749XX
Assault	E9650, E9651, E9652, E9653, E9654, E9794	Y384X1, Y384X2, Y384X3, X93XXX, X940XX, X941XX, X942XX, X948XX, X949XX, X958XX, X959XX
Undetermined	E9850, E9851, E9852, E9853, E9854	Y22XXX, Y230XX, Y231XX, Y232XX, Y233XX, Y238XX, Y239XX, Y248XX, Y249XX
Legal intervention or war	E970	Y35001, Y35002, Y35003, Y35009, Y35011, Y35012, Y35013, Y35019, Y35021, Y35022, Y35023, Y35029, Y35031, Y35032, Y35033, Y35039, Y35091, Y35092, Y35093, Y35099, Y36420, Y36421, Y36430, Y36431, Y3692X, Y37420, Y37421, Y37430, Y37431, Y3792X

Only initial encounters were considered for each diagnosis code under ICD-10-CM

For each year in our sample, we calculated the frequency of firearm hospitalizations for each intent and in total. We then used regression analysis to estimate the average year-over-year change in the frequencies. We did so by taking the natural logarithm of each frequency and regressing it on the year to obtain a coefficient that could be interpreted as a *percent* change. To account for the potential non-comparability of injury surveillance across ICD-9-CM and ICD-10-CM eras, we ran regressions across 2000–2014 and 2016–2020 separately, in addition to across the pooled sample of all years. Estimates from the pooled sample of all years will only be valid if there were no meaningful changes in injury surveillance in the transition from ICD-9-CM to ICD-10-CM.

Next, we calculated the proportion of all inpatient encounters due to a firearm injury each year. We did so by dividing our total annual firearm hospitalization frequencies by total annual inpatient encounters (divided by 100,000). We obtained data on total inpatient encounters from HCUP documentation. We then regressed the rate of firearm hospitalizations per 100,000 inpatient encounters on the year to estimate the average year-over-year change in the rate. Again, we stratified by ICD era and also considered all years together in separate regression analyses.

Finally, we pooled all years in our data and stratified by intent to examine the types of firearms responsible for firearm hospitalizations within each intent. We used the labels/descriptions on ICD codes to elucidate firearm types. Notably, when the intent was legal intervention or war, the single ICD-9-CM code did not specify firearm type, so we were unable to determine firearm type for this intent (for the years 2000–2014). The three types of firearms that we considered (based on the ICD-9/10-CM labels) were (i) handgun, (ii) rifle, shotgun and larger firearm, and (iii) other or unspecified.

## Results

**Fig. 1:**
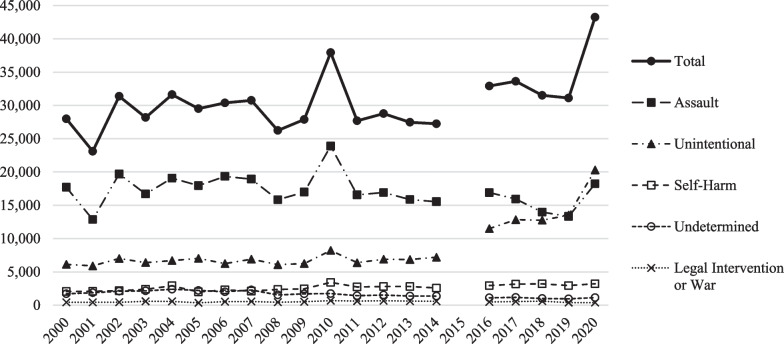
Annual frequency of firearm hospitalizations, by intent. Underlying data came from “Frequencies by Diagnosis and Procedure Codes” released by HCUP from their National Inpatient Sample.  ICD-9-CM codes were used for 2000-2014.  ICD-10-CM codes were used for 2016-2020

From 2000–2020 (excluding 2015), total annual firearm hospitalizations averaged 30,428 per year (Fig. 1). The minimum annual frequency was 23,111 in 2001. The maximum annual frequency was 43,255 in 2020, which was 39% higher than the frequency one year previously in 2019. Each year, with the exception of 2019 and 2020, assault was the most frequent intent. Beginning in 2019, unintentional firearm hospitalizations outnumbered assault firearm hospitalizations.

During the ICD-9-CM era, there was no statistically significant year-over-year change in the frequency of total annual firearm hospitalizations (Table 2, Panel A). There were, however, compositional changes across time with respect to the intent of the shooter. Firearm hospitalizations that were unintentional rose an average of 0.82% each year (*p* = 0.046), firearm hospitalizations in which the intent was self-harm rose an average of 2.20% each year (*p* = 0.001), and firearm hospitalizations in which the intent was legal intervention or war rose an average of 2.85% each year (*p* = 0.000), but firearm hospitalizations with undetermined intent fell an average of 3.02% each year (*p* = 0.005).

**Table 2:** Regression results

	Frequency of firearm hospitalizations						
	Assault Intent	Unintentional intent	Self-harm intent	Undetermined intent	Legal intervention or war intent	Total	Per 100,000 inpatient encounters
	(1)	(2)	(3)	(4)	(5)	(6)	(7)
A. ICD-9-CM era							
Average year-over-year change, 2000–2014	0.03% ↓	0.82% ↑	2.20% ↑	3.02% ↓	2.85% ↑	0.22% ↑	0.20 ↑
	(*p* = 0.977)	(*p* = 0.046)	(*p* = 0.001)	(*p* = 0.005)	(*p* = 0.000)	(*p* = 0.742)	(*p* = 0.673)
*R*-squared	0.0001	0.1854	0.4006	0.4934	0.4406	0.0085	0.0114
Observations	15	15	15	15	15	15	15
B. ICD-10-CM era							
Average year-over-year change, 2016–2020	0.27% ↓	11.89% ↑	1.05% ↑	1.60% ↓	7.72% ↓	4.69% ↑	7.68 ↑
	(*p* = 0.961)	(*p* = 0.071)	(*p* = 0.452)	(*p* = 0.617)	(*p* = 0.161)	(*p* = 0.358)	(*p* = 0.285)
R-Squared	0.0011	0.7288	0.1372	0.0826	0.3724	0.3027	0.3916
Observations	5	5	5	5	5	5	5
Average year-over-year change, 2016–2019	8.45% ↓	4.80% ↑	0.19% ↑	6.23% ↓	5.72% ↓	2.34% ↓	1.85 ↓
	(*p* = 0.007)	(*p* = 0.051)	(*p* = 0.945)	(*p* = 0.093)	(*p* = 0.604)	(*p* = 0.099)	(*p* = 0.096)
R-Squared	0.9661	0.8321	0.0030	0.7313	0.1453	0.6881	0.6846
Observations	4	4	4	4	4	4	4
C. ICD-9-CM & ICD-10-CM eras							
Average year-over-year change, 2000–2014 & 2016–2020	0.64% ↓	4.55% ↑	2.14% ↑	4.04% ↓	0.40% ↑	1.08% ↑	1.32 ↑
	(*p* = 0.274)	(*p* = 0.000)	(*p* = 0.000)	(*p* = 0.000)	(*p* = 0.614)	(*p* = 0.037)	(*p* = 0.039)
R-squared	0.0787	0.6617	0.6175	0.7775	0.0169	0.2525	0.3186
Observations	20	20	20	20	20	20	20
Average year-over-year change, 2000–2014 & 2016–2019	0.88% ↓	3.76% ↑	2.15% ↑	4.18% ↓	0.93% ↑	0.66% ↑	0.70 ↑
	(*p* = 0.155)	(*p* = 0.000)	(*p* = 0.000)	(*p* = 0.000)	(*p* = 0.261)	(*p* = 0.082)	(*p* = 0.016)
R-Squared	0.1277	0.6325	0.5810	0.7662	0.0868	0.1312	0.2142
Observations	19	19	19	19	19	19	19

The above estimates represent coefficients from ordinary least squares regressions. P-values are in parentheses. Standard errors were corrected for heteroscedasticity

Again, during the ICD-10-CM era, there was no statistically significant year-over-year change in the frequency of total annual firearm hospitalizations across the years 2016–2020 (Table 2, Panel B). While there was a marginally significant increase in firearm hospitalizations in which the intent of the shooter was unintentional (11.89% average annual increase, *p* = 0.071), there was no other change in the trend for any other intent across the years 2016–2020.

Because firearm hospitalizations were noticeably higher in 2020, we tested for whether 2020 was an influential point in our regressions. We found that the 2020 observation was an influential point some of the time, so we ran the regressions without 2020 for comparison. Across the years 2016–2019, we found a marginally significant decrease in total firearm hospitalizations (2.34% average annual decrease, *p* = 0.099) driven primarily by a decline in firearm hospitalization with assault intent.

Panel C of Table 2 presents estimates from a linear regression model where data from both the ICD-9-CM and ICD-10-CM eras were pooled. The validity of these results requires comparability of injury surveillance under ICD-9-CM and ICD-10-CM. These results suggest a statistically significant year-over-year increase in the frequency of total annual firearm hospitalizations from 2000 to 2020 (1.08% average annual increase, *p* = 0.037) with a 4.55% average annual increase in unintentional firearm hospitalizations (*p* = 0.000), a 2.14% average annual increase in self-harm firearm hospitalizations (*p* = 0.000), and a 4.04% average annual decrease in firearm hospitalizations with undetermined intent (*p* = 0.000). Results were generally similar in terms of direction and precision when we excluded 2020 from the pooled sample.

**Fig. 2:**
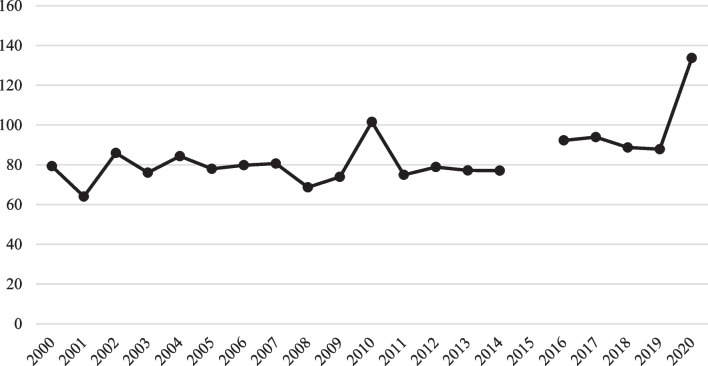
Annual firearm hospitalizations per 100,000 inpatient encounters. For the numerator, underlying data came from “Frequencies by Diagnosis and Procedure Codes” released by HCUP from their National Inpatient Sample.  ICD-9-CM codes were used for 2000-2014. ICD-10-CM codes were used for 2016-2020. For the denominator, underlying data on total inpatient encounters (across all diagnoses) came from HCUP documentation in “Introduction to the HCUP National Inpatient Sample (NIS) 2020”

As a proportion of all inpatient encounters, firearm hospitalizations represented 84 out of every 100,000 inpatient encounters each year, on average, from 2000 to 2020 (Fig. 2). This rate was lowest in 2001 at 64 out of every 100,000 inpatient encounters and highest in 2020 at 134 out of every 100,000 inpatient encounters. During the ICD-9-CM era, there was no statistically significant change in the rate of firearm hospitalizations per 100,000 inpatient encounters (Table 2, last column). During the ICD-10-CM era, we estimated no statistically significant change in the rate of firearm hospitalizations per 100,000 inpatient encounters when 2020 was included, but a marginally significant decline in firearm hospitalizations per 100,000 inpatient encounters when 2020 was excluded. Yet, in the pooled sample of both the ICD-9-CM and ICD-10-CM eras, we estimated increases in the rate of firearm hospitalizations per 100,000 inpatient encounters (1.32 additional firearm hospitalizations per 100,000 inpatient encounters each year, on average, including 2020, *p* = 0.039; 0.70 additional firearm hospitalizations per 100,000 inpatient encounters each year, on average, excluding 2020, *p* = 0.016). Given the estimated effects within each of the individual eras, the overall increase may be attributed to an increase *between* the ICD-9-CM and ICD-10-CM eras. Again, whether a between-era increase is real or artificial depends on the consistency of injury surveillance between eras, an issue beyond the scope of this study.

The composition of the types of firearms responsible for injuries across intents is shown in Table 3. Diagnosis codes indicated that when the intent of the shooter was assault, 27.93% of firearm hospitalizations involved a handgun, 5.87% involved a rifle/shotgun/larger firearm, and 66.20% involved an other/unspecified firearm. When the intent of the shooter was unintentional, 23.94% of firearm hospitalizations involved a handgun, 10.48% a rifle/shotgun/larger firearm, and 65.59% an other/unspecified firearm. When the intent of the shooter was self-harm, 46.63% of firearm hospitalizations involved a handgun, 14.35% a rifle/shotgun/larger firearm, and 39.02% an other/unspecified firearm. Finally, when the intent of the shooter was undetermined, 17.82% of firearm hospitalizations involved a handgun, 6.21% a rifle/shotgun/larger firearm, and 75.96% an other/unspecified firearm. Throughout the ICD-9-CM era, there was a slight shift towards handguns for both unintentional and self-harm firearm hospitalizations; throughout the ICD-10-CM era, there was a shift towards other/unspecified firearms for all intents (excluding legal intervention or war).

**Table 3:** Types of firearms causing hospitalizations, by intent (across all years)

	Assault	Unintentional	Self-harm	Undetermined	Legal intervention or war
Handgun	27.93%	23.94%	46.63%	17.82%	N/A
Rifle, shotgun and larger firearm	5.87%	10.48%	14.35%	6.21%	N/A
Other and unspecified	66.20%	65.59%	39.02%	75.96%	N/A
Number of hospitalizations	342,183	171,026	52,475	32,614	10,269

Underlying data came from “Frequencies by Diagnosis and Procedure Codes” released by HCUP from their National Inpatient Sample. ICD-9-CM codes were used for 2000–2014. ICD-10-CM codes were used for 2016–2020. Firearm type was determined using ICD labels/descriptions

## Discussion

In this study, we used publicly available data from the National Inpatient Sample of HCUP to measure the burden of firearm injuries on the USA hospital system. We leveraged annual frequencies of diagnosis codes (which delineated shooters’ intents and firearm types) for the analysis. We found that firearm injuries inflict a significant burden on the hospital system in the USA, comprising an average of 84 in every 100,000 inpatient encounters each year.

The results revealed that while most firearm hospitalizations have traditionally been due to assault intent, this changed in 2019 when a greater number of firearm hospitalizations were unintentional. This notable increase in accidents suggests the need to address firearm safety. Promoting firearm safety with respect to handguns may be especially warranted as handguns were involved in more injuries resulting in hospitalization than rifles/shotguns/larger firearms across all intents.

Our analysis uncovered no strong evidence of a change in the number of firearm hospitalizations over time in the USA, beginning in 2000 and going through 2020. This result stands in contrast to previous research that reported a slight decline in firearm hospitalization rates from 2000 to 2010 (Kalesan et al. 2014). Yet, our analysis did uncover noticeably more firearm hospitalizations in 2020 than in other years. The year 2020 was unique for a variety of reasons. Among these, Black Lives Matter protests occurred along with COVID-19. It is possible that the unique events of 2020 drove the spike in firearm hospitalizations that we observed in 2020. However, the 2020 spike deserves further investigation.

Our research had several limitations. First, we could not analyze 2015 because annual frequencies of hospitalizations by diagnosis code were not reported by HCUP that year. Second, others have found that firearm injuries increased after the transition from ICD-9-CM to ICD-10-CM (Slavova et al. 2018), so our regression analysis on the pooled sample of 2000–2020 will yield misleading results if the transition created an artificial increase due to coding rather than real-world events. Additionally, changes in coding instructions could have impacted how intents were classified (Barber et al. 2021). It is difficult to assess whether our regression results from the pooled sample provide artificial or real findings. Third, improved completeness of external cause-of-injury codes over the years may have affected the trends we estimated. Fourth, our use of inpatient data prevented us from analyzing visits to hospital-based emergency departments where the patient was discharged from the emergency department or died prior to hospital admission. Fifth, some of the unspecified firearms in our analysis may have been handguns, rifles, shotguns or larger firearms, affecting our ability to precisely describe the composition of firearm types. Sixth, because the use of external cause-of-injury codes in administrative data may not be enforced in all states, there may have been under-reporting of injuries.

Future work should explore how gun regulations have affected the burden of firearm injuries on the hospital system. It would also be informative to explore heterogeneity across states. If particular regions of the USA are experiencing disproportionate burdens on their hospital systems, further exploration may reveal the sources of these burdens.

## Data Availability

The datasets generated during and/or analyzed during the current study are available online from the Healthcare Cost and Utilization Project and can be accessed at the following link: www.hcup-us.ahrq.gov/db/nation/nis/nisdbdocumentation.jsp. We used the Excel files “NIS 2016–2020 with ICD-10-CM/PCS” and “NIS 2000–2014 with ICD-9-CM”, which can be found under the online heading “Frequencies by Diagnosis and Procedure Codes.” Total annual hospitalizations (inpatient encounters) came from Table 3 in Appendix 1 at the following link: https://www.hcup-us.ahrq.gov/db/nation/nis/NISIntroduction2020.pdf. Per HCUP’s recommendation, we used “Number of Discharges in the NIS, Weighted with Trend Weight” for 2000–2011 and “Number of Discharges in the NIS, Weighted” for 2012–2020. The CDC’s External Cause-of-Injury matrices were obtained from the following link: https://www.cdc.gov/nchs/injury/injury_tools.htm.

## Abbreviations

HCUP: Healthcare Cost and Utilization Project
ICD-9-CM: International Classification of Diseases, Ninth Revision, Clinical Modification
ICD-10-CM: International Classification of Diseases, Tenth Revision, Clinical Modification
NIS: National (Nationwide) Inpatient Sample
